# Origin and Development

**Published:** 1894-02-03

**Authors:** 


### THE STUDY OF MAN.

### IV.—ORIGIN AND DEVELOPMENT.

Great as has been the success of recent biological work, in determining the course, and in some instances the causes, of the adaptation of individual members and organs of their special functions, there are still very few cases in which the development of the different species of a whole genus, and their gradual divergence from an archetypal form can be demonstrated with any certainty of detail. Perhaps the best known instance is that of the horse, whose pedigree can be illustrated by a series of fossil forms, chiefly found in the tertiary rocks of America, which show the gradual adaptation of small four-toed and five-toed quadrupeds, with no remarkable running powers, into the finely-built, single-hoofed, and delicately organised animal which we know now.

In the case of man, however, though hypothetical lines of descent have been traced in some detail by various authorities, the question of his origin and nearest connections in the animal kingdom still remains most obscure. For while the gap to be supplied between man and the higher apes of to-day is in some primary characters very considerable, so that the human species has been classed not only in a separate genus, but even by some writers in a distinct order, there is a surprising lack of palæontological material on both sides by which to bridge it over in a historical sense. Human remains of early period are, in particular, extremely rare; and almost without exception they fall still between limits within which the physical structure of man has varied but little. Even the celebrated skull from Neanderthal, though its estimated brain capacity and apparent form place it very low in the series of development, is so little removed from existing human forms that it is said to stand above several recorded skulls of idiots, and has itself been thought by eminent authorities to be an exceptional specimen even for its own period, and therefore not very fair evidence for the average type of man even at that early time. With this exception, in fact, it may be fairly said that the earliest known human skulls do not differ more from those of existing human varieties than the latter do from one another, and the same is true even more emphatically of the other parts of the skeleton.

On the side of the apes the state of the evidence is very similar. The remains which are found in the tertiary strata of South Europe, in particular, and which appear so far back as the miocene series, can be grouped without question very near to their living successors; and the record, though here too far from ample, is sufficiently complete to justify the conclusion that, on this side at all events, the characteristic features of the principal types were established quite early in tertiary time. Either, then, man is a later and very rapidly differentiated offshoot of the anthropoid stock, or else his ancestry runs parallel with, but independent of it, back to a much earlier period, in which case the absence of all trace either of his person or of his industry must be ascribed to the peculiarly imperfect state of the record which geology supplies of the existence and distribution of any land animal, in strong contrast to the abundant evidence which we usually have of marine forms of life.

The alternative, above stated, that man is a late offshoot from the type to which his nearest relations belong, has this advantage, that it points to a peculiarly appropriate period of geological time as the era of his separation from it. The so-called Ice Age, which in the northern hemisphere drove the existing sub-tropical inhabitants of the great land masses uniformly many degrees southward, and exterminated a large number of miocene and pleoscene forms, presented conditions which may be regarded as exceptionally favourable to the development of a species whose supreme characteristic is its power of making itself independent in great measure of external conditions; which clothes and shelters itself against extremes of cold; walks or climbs, in its natural state, with almost equal facility; uses tools to supply its wants, and in particular missile weapons to bring down animal prey at a distance; and by the use of fire, which is unknown even among those apes which have been described as using occasionally both ornaments and missiles, both warms itself, and prepares animal food—a characteristically warming diet—for a digestion which seems, on the evidence of dentition, to have been previously adapted to a vegetarian *régime*. And there is some force in the observation, that the existing races of man in the Old World—America has almost certainly been peopled thence later—seem to radiate, in a manner, from the region of North Africa; the southern part, that is, of the area in which the greater number of tertiary primates occur.

On the other hand, even allowing much for the possibility of very rapid modifications of structure under such exceptional conditions—and we must remember that the period in which we now are is probably equally remarkable for the general stability of organic forms—the glacial period seems almost too close to allow of the establishment of features so distinctive as those, in particular, of the human extremities; of the hand with "opposed" thumb for grasping and climbing, the foot with the great toe so set in line with the rest as to give peculiar strength in springing, and with prominent heel to take the dead weight of the whole body when at rest in the erect position. The last-named character, too, is bound up with a number of structural peculiarities affecting nearly every member of the organism; and in particular, as Dr. Munro has lately pointed out, with the new carriage of the head, necessitated by the enormous development of the cerebral hemispheres, which is the physical representation of man's incomparably superior mental power. The mental power itself, perhaps, is the character which might be acquired in the most rapidly increasing progression, but the skeletal changes above named seem to postulate a period of development far longer than that which is allowed by the chronology of the Glacial Age.

